# Ultrasound-Assisted Extraction and Microencapsulation of *Durvillaea incurvata* Polyphenols: Toward a Stable Anti-Inflammatory Ingredient for Functional Foods

**DOI:** 10.3390/foods14132240

**Published:** 2025-06-25

**Authors:** Nicolás Muñoz-Molina, Javier Parada, Angara Zambrano, Carina Chipon, Paz Robert, María Salomé Mariotti-Celis

**Affiliations:** 1 Graduate School, Faculty of Agricultural and Food Sciences, Universidad Austral de Chile, Isla Teja Campus, Valdivia 5090000, Chile; nico94munoz@gmail.com; 2 Institute of Food Science and Technology, Agricultural and Food Sciences, Universidad Austral de Chile, Isla Teja Campus, Valdivia 5090000, Chile; 3 Institute of Biochemistry and Microbiology, Faculty of Sciences, Universidad Austral de Chile, Isla Teja Campus, Valdivia 5090000, Chile; angara.zambrano@uach.cl (A.Z.); cchipon21@outlook.com (C.C.); 4 Department of Food Science and Chemical Technology, Faculty of Chemical and Pharmaceutical Sciences, Universidad de Chile, Santos Dumont 964, Independencia, Santiago 8380000, Chile; proberts@uchile.cl; 5 Nutrition and Dietetics School, Faculty of Medicine, Universidad Finis Terrae, Pedro de Valdivia 1509, Providencia, Santiago 7501015, Chile; mmariotti@uft.cl

**Keywords:** *Durvillaea incurvata*, seaweed polyphenols, microencapsulation, anti-inflammatory foods, functional ingredients

## Abstract

*Durvillaea incurvata*, a Chilean brown seaweed, exhibits high antioxidant activity and polyphenol content, positioning it as a promising candidate for developing bioactive food ingredients. This study evaluated the anti-inflammatory activity of an ethanolic extract of *Durvillaea incurvata*, produced via ultrasound-assisted extraction, and its subsequent microencapsulation to obtain a functional food-grade ingredient. The extract’s anti-inflammatory capacity was assessed in vitro through hyaluronidase inhibition, and its cytotoxicity was evaluated using gastrointestinal cell models (HT-29 and Caco-2). Microencapsulation was performed by spray-drying with maltodextrin, and encapsulation efficiency (EE) was optimized using response surface methodology. Characterization included scanning electron microscopy, differential scanning calorimetry, and X-ray diffraction. The extract exhibited low cytotoxicity (cell viability > 75%). Optimal encapsulation conditions (inlet temperature: 198.28 °C, maltodextrin: 23.11 g/100 g) yielded an EE of 72.7% ± 1.2% and extract recovery (R) of 45.9% ± 2.4%. The microparticles (mean diameter, 2.75 µm) exhibited a uniform morphology, shell formation, glassy microstructure, and suitable physicochemical properties (moisture, 3.4 ± 0.1%; water activity, 0.193 ± 0.004; hygroscopicity, 30.3 ± 0.4 g/100 g) for food applications. These findings support the potential of microencapsulated *Durvillaea incurvata* extract as an anti-inflammatory ingredient for functional food development.

## 1. Introduction

Seaweed represents a rich and underexploited source of bioactive compounds with potential applications in nutraceuticals, pharmaceuticals, and functional foods. Among marine macroalgae, brown seaweeds (Phaeophyceae) are particularly notable due to their high content of polyphenols—mainly phlorotannins—alongside complex polysaccharides and minerals [1].

Nowadays, the anti-inflammatory role of dietary polyphenols is a topic that has gained significant attention. Polyphenols target multiple inflammatory components and lead to anti-inflammatory mechanisms, regulating immunity by interfering with immune cell regulation, proinflammatory cytokine synthesis, and gene expression [2]. In general, a high dietary polyphenols intake is associated with a decreased risk of chronic diseases related to inflammation, and although the mechanisms responsible for their anti-inflammatory effect in vivo are still not fully understood, there have been identified at least eight mechanisms: direct antioxidant effect, indirect antioxidant effect, inhibition of pro-inflammatory mediators, intestinal barrier protection, prebiotic effects, inhibition of digestive enzymes, metabolic effects, and regulation on protein kinases [3].

Despite this, most bioactivity-focused studies have concentrated on a limited number of genera, and significant gaps persist in early-stage evaluations of potential anti-inflammatory properties, as well as in the technological development of functional ingredients, particularly from endemic species such as *Durvillaea incurvata*, which remains underexplored despite its promising bioactive profile.

*Durvillaea incurvata*, commonly known as “cochayuyo” in Chile, is a brown seaweed endemic to the southeastern Pacific and traditionally consumed along the Chilean coast. Its documented antioxidant, anti-inflammatory, lipid-lowering, and anti-obesity properties highlight its potential as a source of bioactive compounds for functional applications [4].

However, translating these biological effects into viable, functional food ingredients requires the development of scalable extraction and stabilization strategies [5]. Among these, ultrasound-assisted extraction has emerged as a green and efficient technique that enhances the recovery of thermolabile compounds by promoting cell disruption and solvent penetration, thereby reducing both solvent consumption and processing time [6]. Previous work by our group demonstrated that UAE applied to *Durvillaea incurvata* produced extracts with superior antioxidant capacity and enzyme-inhibitory activity compared to conventional extraction methods [7].

To complement these extraction advances, reliable bioactivity screening methods are also essential. Given the high cost and complexity of in vivo assays, in vitro enzymatic tests—such as hyaluronidase inhibition—offer a practical and informative approach for early-stage evaluation of anti-inflammatory potential. This method facilitates the identification of promising candidates prior to engaging in more resource-intensive mechanistic or clinical assessments. While additional assays will be necessary to confirm efficacy under physiological conditions, hyaluronidase inhibition provides a valuable first-line indicator of anti-inflammatory activity at the laboratory scale.

Building on this initial screening, further formulation steps are required to ensure the stability and functionality of the extract in real food systems. Although polyphenolic extracts exhibit promising biological properties, their susceptibility to heat, light, oxygen, and gastrointestinal conditions poses significant challenges for incorporation into food matrices [8]. To address this limitation, microencapsulation—particularly through spray-drying—has been extensively employed in the food industry to improve compound stability, protect bioactivity, and enable controlled release [9]. Maltodextrin is frequently selected as a wall material due to its high water solubility, low viscosity, and compatibility with food-grade applications, being then used to microencapsulate several bioactive compounds, where microparticles obtained by spry-drying have shown suitable yield, good encapsulation efficiency, a glassy microstructure, and a spherical particle shape with a smooth surface, suggesting good stability [10,11,12,13]. Moreover, encapsulation enhances dispersibility and can mitigate sensory drawbacks commonly associated with phenolic-rich ingredients [14].

To optimize the encapsulation process and ensure efficient use of materials and energy, response surface methodology (RSM) offers a powerful statistical tool. RSM allows for the simultaneous evaluation of multiple variables and their interactions, enabling the identification of optimal conditions with a reduced number of experimental trials. It is widely applied in food engineering to model complex processes, such as encapsulation efficiency, antioxidant retention, and bioactive protection [15]. In this study, we aimed to (1) obtain a polyphenol-rich extract of *Durvillaea incurvata* using UAE and evaluate its anti-hyaluronidase activity and cytotoxicity in gastrointestinal cell models; and (2) encapsulate the extract using spray-drying with maltodextrin as the wall material, optimizing the process via RSM. This study provides foundational evidence supporting the stabilization and in vitro evaluation of bioactive compounds from seaweed, offering a relevant basis for their potential incorporation into food systems. The insights generated here are expected to guide future work focused on in vivo validation and food matrix integration, facilitating the development of functional ingredients for health-promoting applications.

## 2. Materials and Methods

### 2.1. Materials

The seaweed *Durvillaea incurvata* was collected from the “Palo Muerto” sector (Latitude: −39.8833° Longitude: −73.5167°), Corral commune, Chile. Immediately, it was cleaned with seawater and stored in a box to protect it from light during transport to the lab (all done within 24 h). In the laboratory, the seaweed was washed with distilled water, cut into cubes of approximately 1 cm^3^, freeze-dried, and ground to a particle size of approximately 0.5 mm. Samples were then stored (−80 °C) until extraction.

All chemicals and reagents were of analytical grade. Unless stated otherwise, most of them were acquired from Sigma Chemical Co. (Saint Louis, MO, USA).

### 2.2. Ultrasound-Assisted Extract (UAE) Obtention

The extraction was carried out according to the optimal conditions obtained by Muñoz-Molina et al. [7]. Briefly, extraction using 70% ethanol (*v*/*v*) was carried out for 80 min at 50 °C using a thermoregulated bath without agitation. An ultrasonic processor (Sonics VCX series, 500 W, 20 kHz, Sonics & Materials Inc., Newtown, CT, USA) was used to generate ultrasonic waves every 8 s, stimulating extraction. Once the extraction was finished, the extract was filtered with a 0.45 μm cellulose syringe filter and stored at −80 °C until use.

### 2.3. Cytotoxicity Assessment of UAE

To corroborate its safety as a food ingredient, the cytotoxicity of UAE was assessed based on the method described by Pacheco et al. [16]], using HT-29 and Caco-2 cells. The propidium iodide (PI) viability assay was performed in 96-well microplates. For each well, 5000 cells (HT-29 or Caco-2) were seeded per well in a volume of 100 µL of RPMI-1640 medium without phenol red, supplemented with 10% fetal bovine serum and 1% penicillin-streptomycin solution. The cells were allowed to acclimatize until the next day to allow their adhesion to the plate. Subsequently, the cells were treated with different concentrations of the extract (100, 500, 750, and 1000 µg/mL). Two µL of dimethylsulfoxide (DMSO) was used as a negative control, and 100 µL of dimethylformamide (DMF) was used as a positive one. The effects of the treatments on cell viability were evaluated at 24 and 48 h. Once the treatment times were completed, 100 µL of HBSS-Ca2+/PI buffer was added to the wells corresponding to the treatments and negative control, and 2 µL of 500 µM propidium iodide was added to the wells corresponding to the positive control, thus achieving the same final concentration of propidium iodide in all wells. The plate was incubated at 37 °C for 5 min, and propidium iodide incorporation into the cells was measured by determining the fluorescence with an emission wavelength of 530 nm and an excitation wavelength of 620 nm in a Varioskan^®^ Flash multiple reader (Thermo Fisher Scientific Inc., Waltham, MA, USA). Finally, the percentage of cell viability was calculated with respect to the negative control (average of 3 different experiments).

### 2.4. Hyaluronidase Inhibitory Activity of UAE

The assay was performed according to the protocol described by Ling et al. [17] with slight modifications. First, assay medium (1.00–1.67 U hyaluronidase in 95 μL 20 mM sodium phosphate buffer pH 7.0 with 77 mM sodium chloride and 0.01% BSA) was preincubated with 5 μL of the extract (in DMSO) for 10 min at room temperature (5 μL DMSO without extract were used as control). After that, 100 μL hyaluronic acid (0.03% *w*/*v* in 300 mM sodium phosphate, pH 5.35) was added, and the mixture was incubated for 45 min at 37 °C. Then, 1 mL acid albumin solution (0.1% bovine serum albumin in 24 mM sodium acetate and 79 mM acetic acid, pH 3.75) was added, and the mixture was incubated for 10 min at room temperature. Finally, the absorbance was measured at 600 nm and the inhibitory activity of UAE was calculated using Equation (1):

(1) $$\%\;Inhibition=\frac{\left(A_{x}-A_{c}\right)}{A_{c}}\times 100$$

where *A_x_* is the absorbance of the extracted sample, and A_c_ is the absorbance of the control (DMSO, does not have extract).

The extract was tested at a maximum concentration of 150 μg/mL in the final reaction mixture, and the IC_50_ value was calculated. The experiment was performed in triplicate and the results expressed as the mean ± standard deviation.

### 2.5. Microencapsulation of UAE

The encapsulation of UAE was performed by spray-drying using a B-290 mini spray-dryer (Büchi, Switzerland) and maltodextrin (MD) as the encapsulating agent.

Briefly, 20 g of crude UAE was mixed at room temperature with MD at different concentrations and water (to make 100 g). The mixture was then homogenized under magnetic stirring for 25–30 min before spray-drying. Response surface methodology (RSM) was used to optimize the process, and since the independent variables are independent of each other, central composite design (with four central points) was used [15]. The independent variables (see Table 1) were the inlet temperature (x_1_, 150 to 190 °C) and the coating agent concentration in the feeding (x_2_, 5 to 20%). The extract concentration was set at 20 g/100 g for all samples. The dependent variable to be maximized was the encapsulation efficiency (EE). Recovery (R) was also measured, and the constant parameters were air flux, 600 L/h; feeding rate, 1 mL/min; and atomization pressure, 0.5 MPa. The resulting powders were weighed and kept at 4 °C in closed bags protected from light until analysis. The optimal spray-drying conditions were obtained using a second-order model (Equation (2)) with the Statgraphics Centurion XVI software package.

(2) $$y=\beta_{0}+\sum_{i=1}^{k}\beta_{i}X_{i}+\sum_{i=1}^{k}\beta_{ii}X_{ii}^{2}+\sum_{i=1}^{k-1}\sum_{j=2}^{k}\beta_{ij}X_{i}X_{j}+E$$

where X refers to the independent variables, whereas β refers to the regression coefficients (obtained using the method of least squares), y is the encapsulated efficiency (EE), and E is the residual error.

### 2.6. Microparticle Analysis

All of the following analyses were performed on the powder obtained under the optimal microencapsulation conditions defined above.

#### 2.6.1. Encapsulation Efficiency and Recovery

##### Total Polyphenols

To determine total polyphenols, the structure of the microcapsule coating material was completely broken by mixing 100 mg of microcapsules and 2 mL of methanol/acetic acid/water (50:8:42 *v*/*v*/*v*), followed by dissolution by vortexing (1 min), ultrasonication for 30 min, and finally centrifugation at 9000 rpm for 5 min. The phenolic compounds in the supernatant were measured according to the Folin–Ciocalteu colorimetric method [18] and expressed in µg of gallic acid equivalent (GAE) (calibration curve: 1.25–20 µg/mL; R^2^ = 0.9994).

##### Surface Polyphenols

Four hundred milligrams of microparticles was dispersed in methanol (2 mL) with soft stirring. The dispersion was centrifuged at 2000 rpm for 5 min. The supernatant was considered to contain polyphenols from the surface fraction of the microparticles (${PF}_{Surface}$). The phenolic compounds were measured according to the Folin–Ciocalteu method, as described previously.

Encapsulation efficiency (EE) and recovery (R) were calculated according to the following equations:

(3) $$EE\left(\%\right)=\frac{{PF}_{Total}-{PF}_{Surface}}{{PF}_{Total}}\times 100$$

(4) $$R\left(\%\right)=\frac{{PF}_{Total}}{{PF}_{Total\;theorical}}\times 100$$

where ${PF}_{Total}$ corresponds to total polyphenol content in the microparticle powder (µg GAE/g), ${PF}_{Surface}$ corresponds to the polyphenol content in the surface fraction of the microparticle powder (µg GAE/g), and ${PF}_{Total\;theorical}$ corresponds to the theoretical content of polyphenols in the powder (µg GAE/g), which agreed with the feed solution content.

#### 2.6.2. Moisture Content

The moisture content of the microparticle powder was determined by gravimetry [19]. One gram of microparticles was weighed, placed on a previously weighed Petri dish, and dried in an oven (BE 500, Memmert ^®^, Schwabach, Germany) at 105 °C for 5 h (until constant weight).

#### 2.6.3. Hygroscopicity

The powder’s hygroscopicity was determined by gravimetry as follows: about 0.5 g of microparticles was weighed and distributed in watch glasses and placed in a desiccator with a saturated solution of Na_2_SO_4_ (81% RH) for one week (at room temperature). Hygroscopicity was expressed as g H_2_O/100 g of dry solids [20].

#### 2.6.4. Microparticle Size and Shape

Particle size was measured using a laser scattering particle size distribution analyzer (Partica LA-960, Horiba Scientific, Japan; 650 nm laser diode), where the microparticles to be analyzed were dispersed in ethanol.

Also, microparticle morphology was analyzed using an environmental scanning electron microscope (model EVO MA10, Carl Zeiss, UK), as described previously [21,22], with slight modifications. To study the size and shape of the microparticles, the powder was placed on a sample holder with the help of adhesive double-sided carbon tape, then coated with a thin layer of gold using a vacuum coater (EM ACE200, Leica Microsystems, Wetzlar, Germany) and observed under high-vacuum conditions, with a secondary electron detector (SE1) and an acceleration voltage of 20 kV. An fracture was induced in the microparticles was applied to determine the thickness of the shell of the microparticles. The powder was first placed on copper tape fixed to a specimen slide. Then, the tape was stuck and unstuck by using a second piece of copper tape, achieving a fracture. Finally, samples were coated with gold and observed as described previously. Fractured particles were localized, and the shell thickness was measured. All images were saved in TIFF format (1024 × 768 pixels) and analyzed using ImageJ software (ImageJ 1.54g, National Institute of Health, Bethesda, MD, USA, https://imagej.net).

#### 2.6.5. Differential Scanning Calorimetry

The glass transition temperature (*T*_g_), melting temperature (*T*_m_), and enthalpy of melting (Δ*H*_m_) were analyzed using a differential scanning calorimeter (TA Q20, TA Instruments, New Castle, DE, USA) [23]. Samples (5–10 mg) were placed in sealed aluminum pans (TA T-Zero capsules) and then loaded onto the equipment. An empty sealed aluminum pan was used as a reference in each analysis and nitrogen was used as a carrier gas (50 mL/min). Thermal analysis was performed from −40 °C to 250 °C at a rate of 10 °C/s. The thermal properties of the samples were analyzed from the resulting heat flow thermograms, using TA Instruments Universal Analysis 2000 software (New Castel, DE, USA). The temperature and heat flow were calibrated using indium and distilled water.

#### 2.6.6. X-Ray Diffraction

X-ray diffractometry was used to corroborate the absence of crystalline structures in the microparticles. A SAXSPoint 2.0 instrument (Anton Paar, Austria) was used with a copper X-ray source (Primus 100, CuKα = 1.54178 Å, 50 kV and 100 μA) and a two-dimensional Eiger R 1 M detector (Dectris, Baden-Daettwil, Switzerland). The microparticle sample was placed between Mylar and Kapton sheets. The diffractograms were obtained at 25 °C, 900 s measurement time, and 115 mm distance between the sample holder and detector.

### 2.7. Statistical Analysis

For response surface methodology (RSM), STATGRAPHICS Centurion XV software, version 15.2.06 (Old Tavern Rd, The Plains, VA, USA) was used (95% level of confidence). For any means of comparison, data (triplicate) were analyzed by analysis of variance (ANOVA) followed by Tukey’s multiple comparison test (*p* < 0.05) using the same software (STATGRAPHICS).

## 3. Results and Discussion

### 3.1. Cytotoxicity of UAE

**Figure 1:**
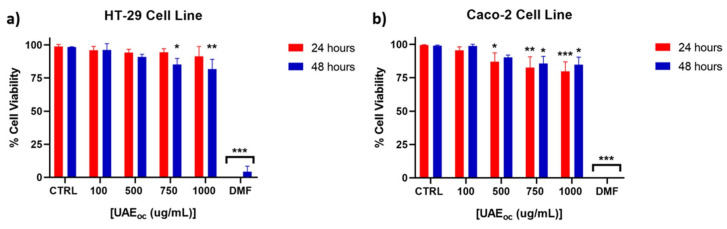
Viability of HT-29 (**a**) and Caco-2 (**b**) cell lines in the presence of cochayuyo extract after 24 and 48 h at increasing concentrations (n = 3). UAE_OC_: ultrasound assisted extract under optimal conditions of extraction; CTRL: control without extract; DMF: dimethylformamide, positive toxicity control. Each bar was compared with CTRL: (*) *p* < 0.05, (**) *p* < 0.01, (***) *p* < 0.001.

The cytotoxicity of the ultrasound-assisted extract (UAE) was assessed using HT-29 and Caco-2 human epithelial cell lines. As shown in Figure 1, cell viability was maintained above the generally accepted cytotoxicity threshold of 75% across most tested concentrations.

Both HT-29 and Caco-2 cell lines are widely employed in in vitro models for intestinal bioavailability and safety evaluation. HT-29 cells represent a model with absorptive and mucus-secreting features, while Caco-2 cells differentiate into enterocyte-like monolayers and are commonly used to simulate intestinal epithelium [24].

For HT-29 cells (Figure 1a), viability remained unaffected at lower concentrations and declined significantly only at 750 µg/mL after 48 h. A similar trend was observed for Caco-2 cells (Figure 1b), where significant reductions began at 500 µg/mL. These concentration-dependent effects suggested a relatively low cytotoxic potential, consistent with safety requirements for food-grade bioactives. These findings are in line with Lordan et al. [25], who observed that brown seaweed extracts reduced cell viability only at concentrations ≥ 1000 µg/mL.

Pacheco et al. [16] evaluated the cytotoxicity of ethanolic and acetone extracts (1–1000 µg/mL) from various seaweed species, including *Durvillaea incurvata*, using the HT-29 cell line, and reported no significant reduction in cell viability. The divergence from our findings—where reduced viability was observed at higher concentrations after 48 h—may be attributed to differences in extraction methodology. Specifically, Pacheco et al. employed high-pressure liquid extraction (HPLE), a technique that likely yields extracts with lower mannitol content and a distinct polyphenol composition compared to those obtained via ultrasound-assisted extraction [5,26]. As noted by Quitério et al. [27], extraction parameters can significantly alter the qualitative and quantitative profile of phenolic compounds. Supporting this, it has been shown that UAE produces a phenolic fingerprint distinct from that of HPLE, which may explain the higher cytotoxicity observed at elevated concentrations in our study [5].

Regarding the Caco-2 cell line, our results are consistent with those of Lordan et al. [25], who reported that aqueous and ethanolic extracts from five brown algae species reduced cell viability only at concentrations of 1000 µg/mL, with no effects observed at lower levels (0.1–100 µg/mL). These findings suggest that the cytotoxic effects of seaweed extracts may be concentration-dependent and linked to the abundance and structure of phenolic compounds. Moreover, since both HT-29 and Caco-2 cells originate from colorectal carcinomas, the observed reduction in viability at higher concentrations could reflect potential antiproliferative effects relevant to cancer prevention or therapy rather than cytotoxicity to normal cells.

### 3.2. Inhibition of Hyaluronidase by UAE

**Figure 2:**
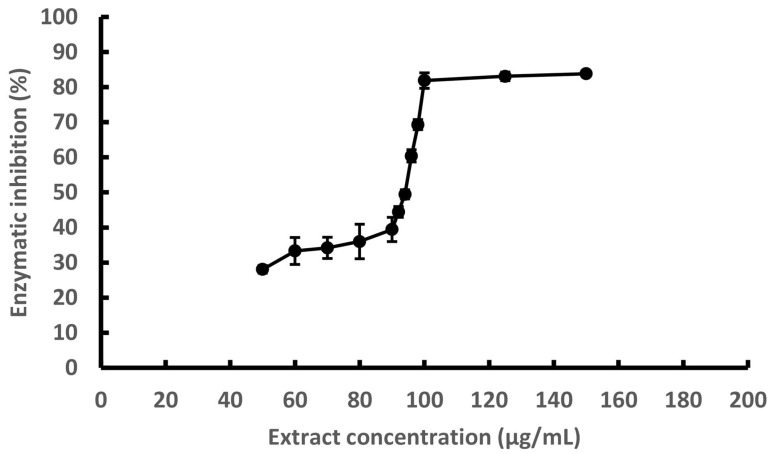
Inhibitory activity of UAE against hyaluronidase enzyme, expressed as inhibition percentage. Each point is the average of three independent samples ± standard deviation.

The inhibitory effect of the UAE on hyaluronidase activity was evaluated for the first time and is presented in Figure 2. The extract exhibited a dose-dependent inhibition, with an IC_50_ value of 93.6 μg/mL and maximal inhibition exceeding 80% at concentrations above 100 μg/mL. This activity is likely attributable to polyphenolic compounds commonly found in brown algae, such as phlorotannins (e.g., phloroglucinol, eckol, phlorofucofuroeckol A, dieckol, and 8,8′-bieckol), which have demonstrated affinity for hyaluronidase and are proposed to act via competitive inhibition mechanisms. Previous studies have also suggested that phlorotannins with higher molecular weights tend to exert stronger inhibitory effects on this enzyme [28,29].

Hyaluronidase catalyzes the depolymerization of hyaluronic acid, a key glycosaminoglycan of the extracellular matrix, and plays a role in inflammation, cancer progression, and allergic reactions [30]. The inhibition of this enzyme has been associated with anti-inflammatory and antiallergic effects and is frequently used as an in vitro proxy for preliminary anti-inflammatory screening. In this context, hyaluronidase inhibition allows for rapid, cost-effective evaluation of anti-inflammatory potential at the laboratory scale, enabling the identification of promising extracts before advancing toward more complex mechanistic or in vivo studies, always taking into account that edible polyphenols could have several other mechanisms of anti-inflammatory action [3].

Comparable inhibitory effects have been observed in other brown seaweed species. For instance, it was reported that ethanolic extracts of *Scytosiphon* sp. exhibited anti-hyaluronidase activity with an IC_50_ of 0.67 mg/mL, which was superior to the conventional antiallergic drug disodium cromoglycate (IC_50_ = 1.13 mg/mL) and similar to epigallocatechin gallate (EGCG, IC_50_ = 0.56 mg/mL), a well-known polyphenolic compound [31].

Although the present study did not include compound-level identification, previous reports indicate that *Durvillaea incurvata* extracts contain multiple bioactives beyond phlorotannins, including alginates, fucoidans, and carotenoids such as fucoxanthin, which have also been linked to anti-inflammatory mechanisms [32,33]. Further research is needed to elucidate the specific compounds responsible for the observed effects and their interactions with hyaluronidase.

In light of the broader biological activities described for *Durvillaea incurvata*, including anti-diabetic, neuroprotective, and antihypertensive potential [7], this species remains a promising candidate for developing functional ingredients. However, a more comprehensive evaluation involving mechanistic cellular assays (e.g., cytokine modulation, NF-κB pathway inhibition), bioaccessibility under gastrointestinal conditions, and compound characterization will be required to validate its application in food and health contexts fully.

### 3.3. Optimization of UAE Microencapsulation Conditions

To improve the stability and potential applicability of the UAE as a bioactive food ingredient, microencapsulation via spray-drying was optimized using response surface methodology (RSM). This statistical approach enables modeling nonlinear interactions between process variables and allows for efficient experimental design in complex systems. In this case, RSM was employed to evaluate the effects of two critical parameters—inlet temperature (IT) and maltodextrin concentration (MD)—on encapsulation efficiency (EE), defined as the percentage of total polyphenols successfully retained within the microparticles.

Table 2 summarizes the EE results for each experimental run in the central composite design. A second-order polynomial regression model was fitted to the data, yielding the following equation:

EE = 960.721 − 11.059 × IT + 2.24 × MD + 0.0325316 × IT^2^ + 0.0035 × IT × MD − 0.0482235 × MD^2^

This model showed a high coefficient of determination (R^2^ = 0.9555; adjusted R^2^ = 0.9183), indicating an adequate fit and predictive capability. ANOVA confirmed the model’s significance (*p* < 0.01), validating its use in identifying optimal encapsulation conditions.

**Figure 3:**
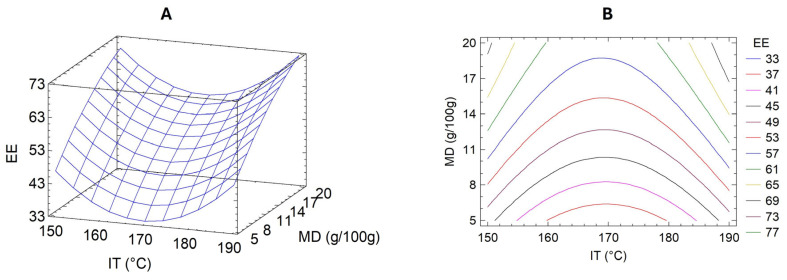
Response surface (**A**) and contour (**B**) plots showing the effects of the inlet temperature (IT) during spray-drying and maltodextrin concentration (in the feeding; MD) on the EE (%) of *Durvillaea incurvata* extract.

The response surface and contour plots (Figure 3) indicated that both low and high inlet temperatures produced relatively high EE values, suggesting a nonlinear relationship between thermal input and polyphenol retention. This outcome may reflect the complex balance between drying kinetics, solvent evaporation rates, and thermal degradation. High temperatures may enhance droplet drying and encapsulation shell formation, minimizing polyphenol losses due to surface exposure. However, excessive heat may degrade sensitive compounds, consistent with the observed variability at the upper-temperature range.

The optimal predicted encapsulation conditions were IT = 198.28 °C and MD = 23.11 g/100 g, with a projected EE of 88.97%. Validation experiments under these conditions yielded an actual EE of 72.7% ± 1.2%, which, although lower than predicted, is consistent with prior findings for polyphenol encapsulation using maltodextrin [10]. These results confirmed the robustness of the model while highlighting inherent process variability, likely due to polyphenol volatility and heterogeneity of extract composition.

In addition to EE, extract recovery (R)—defined as the ratio of total phenolic content in the powder to the amount initially fed into the dryer—was 45.9% ± 2.4% under optimal conditions. This value aligns with previous reports where brown algae extracts were encapsulated using maltodextrin, obtaining R values between 32% and 50% [34]. Lower recovery rates are typically associated with forming fine particles prone to sticking on chamber walls or escaping collection systems.

This study’s RSM model prioritized encapsulation efficiency (EE) as the primary response variable, offering a valuable framework for optimizing spray-drying conditions. While effective for evaluating the influence of inlet temperature and maltodextrin concentration, future work would benefit from a multi-response optimization approach. Including additional criteria such as antioxidant capacity, retention of hyaluronidase inhibition, and process cost efficiency would enable a more comprehensive assessment of encapsulation performance and enhance its industrial relevance.

### 3.4. Microparticles Analysis

The microparticles produced under optimal spray-drying conditions exhibited physicochemical properties consistent with stable formulations suitable for storage and handling. The resulting powder showed a moisture content of 3.4 ± 0.1%, water activity of 0.193 ± 0.004, and hygroscopicity of 30.3 ± 0.4 g/100 g. Particle size analysis revealed an average diameter of 19.17 ± 2.79 μm, falling within the typical range for food-grade microencapsulated products. These values are comparable to those reported for maltodextrin-based seaweed microparticles and suggest good physical stability under appropriate storage conditions [34]. Low water activity (<0.3) is particularly relevant for preventing microbial growth and maintaining the functional integrity of encapsulated bioactives. The hygroscopicity value indicates moderate moisture affinity, which supports the need for proper packaging to avoid clumping or degradation during storage. Particle size and morphology, in turn, influence powder dispersibility, solubility, and reconstitution behavior—key parameters for future incorporation into food systems [35].

#### 3.4.1. Microparticle Morphology by Scanning Electron Microscope

**Figure 4:**
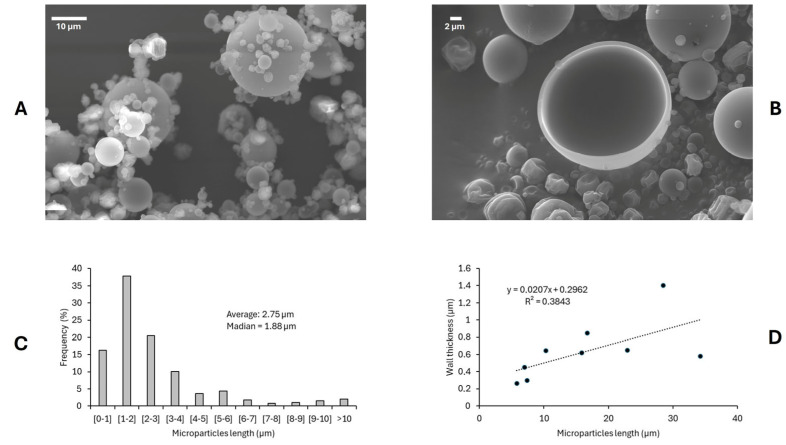
Scanning electron microscopy images of spray-dried particles obtained under optimal condition of encapsulation. (**A**) Intact particles showing the size, shape, and surface; (**B**) Fractured particle showing the particle shell; (**C**) Particle size distribution; (**D**) Wall thickness and particle size relationship.

The microparticles’ morphology under optimal encapsulation conditions was analyzed using scanning electron microscopy. Representative micrographs (Figure 4A) revealed that the spray-dried particles exhibited predominantly spherical shapes with smooth surfaces and minimal visible fissures, which indicated effective encapsulation. The absence of structural collapse and the presence of intact spherical morphology suggested that the maltodextrin concentration and inlet temperature selected were appropriate for microcapsule formation [36].

Agglomeration was observed to be limited and primarily occurred around larger particles, likely due to interactions during particle recirculation in the drying chamber. Although often considered undesirable, controlled agglomeration may improve solubility and dispersion by enhancing wettability and preventing the segregation of powder components [37].

The particle size distribution, presented in Figure 4C, showed that approximately 80% of particles measured less than 4 µm in diameter, with a mean diameter of 2.75 µm and a median of 1.88 µm. Despite most falling within the sub-10 µm range, some particles reached sizes greater than 30 µm. This size distribution aligns with previous studies using maltodextrin for seaweed extract encapsulation, and it has significant implications for powder solubility and bulk properties [34].

A transparent encapsulation shell was observed in fractured particles (Figure 4B), with a wall thickness ranging from ~0.2 µm to ~1.4 µm. Wall thickness tended to increase with particle size. Still, it plateaued around 1 µm for particles larger than 15 µm (Figure 4D). These results confirmed the successful formation of microcapsules and are consistent with prior reports using maltodextrin as wall material [22].

Together, these findings reinforce the adequacy of maltodextrin for stabilizing ultrasound-assisted seaweed extracts.

#### 3.4.2. Differential Scanning Calorimetry of the Microparticles

**Figure 5:**
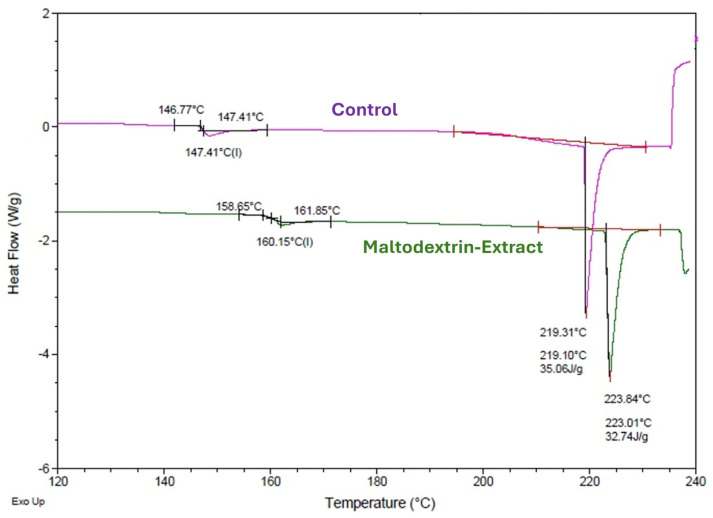
Differential scanning calorimetry thermogram of maltodextrin microparticles having UAE (green). “Control” corresponds to microparticles having only maltodextrin (purple).

Differential scanning calorimetry thermograms (Figure 5) of microcapsules with and without extract showed no thermal transitions below 100 °C, indicating physical stability under typical storage conditions. A glass transition temperature (Tg) of 160.15 °C for the encapsulated sample was recorded, which was higher than that of empty maltodextrin particles. This increase suggested molecular interactions between extract constituents and maltodextrin, such as hydrogen bonding or increased cross-linking density, contributing to structural rigidity [38].

An endothermic peak at approximately 220 °C, attributed to the melting and gelatinization of maltodextrin, was also observed. The higher temperature of this transition in the encapsulated sample reinforced the notion of improved thermal stability conferred by the interaction between polyphenolic compounds and the wall material [39].

#### 3.4.3. X-Ray Diffraction of the Microparticles

**Figure 6:**
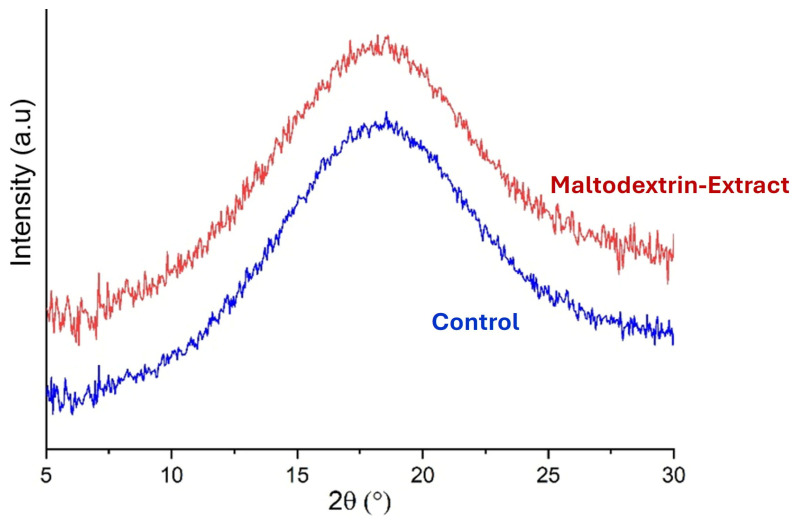
X-ray diffractometry spectra of maltodextrin microcapsules having UAE (red). “Control” corresponds to microparticles having only maltodextrin (blue).

X-ray diffraction analysis (Figure 6) confirmed the amorphous nature of the spray-dried microparticles. Both extract-loaded and empty microparticles showed broad halos without distinct crystalline peaks, suggesting molecular-level dispersion of the bioactive compounds within the maltodextrin matrix. This amorphous state is desirable, as it enhances solubility and facilitates controlled release in aqueous systems [40,41]. The absence of crystallinity also implied that the encapsulation process did not induce phase separation or aggregation of the polyphenols. This further supported the suitability of maltodextrin for stabilizing bioactive seaweed extracts.

## 4. Conclusions

The UAE of *Durvillaea incurvata* yielded a polyphenol-rich extract that exhibited no cytotoxic effects in HT-29 and Caco-2 cell lines at concentrations up to 750 μg/mL, supporting its preliminary safety for food-related applications. In addition, the extract showed significant in vitro anti-inflammatory potential through hyaluronidase inhibition, reinforcing its promise as a bioactive candidate for further investigation. To enhance its stability and applicability, the extract was successfully microencapsulated using spray-drying with maltodextrin, producing microparticles with favorable morphological, thermal, and structural properties. The combination of spherical shape, low water activity, amorphous matrix, and increased glass transition temperature indicated improved compound protection and suitability for incorporation into food systems. Collectively, these findings support the feasibility of using UAE and encapsulation as sustainable strategies to develop stabilized seaweed-derived ingredients, paving the way for future studies on bioavailability, gastrointestinal stability, and application in functional food matrices.

## Figures and Tables

**Table 1:** Independent variables and their ranges for microencapsulating UAE by spray-drying using central composite design.

Independent Variable	Unit	Symbol	Coded and Actual Levels of Factors				
			−1.41	−1	0	+1	1.41
Inlet temperature (IT)	°C	x_1_	141.7	150.0	170.0	190.0	198.3
Maltodextrin concentration (MD)	g/100 g	x_2_	1.9	5.0	12.5	20.0	23.1

**Table 2:** Encapsulation efficiency (EE) of UAE optimized by response surface methodology.

Run	Inlet Temperature (°C)	Maltodextrin Concentration (g/100 g)	EE (%)
1	150.00	5.00	43.3 ± 4.9
2	190.00	5.00	43.2 ± 4.0
3	150.00	20.00	69.0 ± 1.5
4	190.00	20.00	71.0 ± 1.9
5	141.72	12.50	75.3 ± 0.8
6	198.28	12.50	79.1 ± 1.4
7	170.00	1.89	30.1 ± 2.5
8	170.00	23.11	61.4 ± 0.4
9	170.00	12.50	52.7 ± 1.7
10	170.00	12.50	42.8 ± 0.4
11	170.00	12.50	48.5 ± 3.6
12	170.00	12.50	51.0 ± 1.0

## Data Availability

The original contributions presented in this study are included in the article. Further inquiries can be directed to the corresponding author.
